# Retraction Note: Fabrication of a microfluidic device for studying the in situ drug-loading/release behavior of graphene oxide-encapsulated hydrogel beads

**DOI:** 10.1186/s40824-018-0125-y

**Published:** 2018-05-18

**Authors:** Sarath Chandra Veerla, Da Reum Kim, Sung Yun Yang

**Affiliations:** Department of Organic Materials Engineering, Chungnam National University, 99, Daehak-ro, Yuseong-gu, Daejeon 34134 Korea

## Correction

This article [1] has been retracted by the authors due to lack of permission to use and publish the data reported. All authors agree to this retraction.
